# Luspatercept: From Bench to Bedside and Beyond in the Management of Ineffective Erythropoiesis

**DOI:** 10.14740/jh2163

**Published:** 2026-04-06

**Authors:** Dhara Popat, Rajan Desai, Sheikh Abdullah, Siddhant Jain, Poornima Ramadas

**Affiliations:** a Department of Internal Medicine, Louisiana State University Health Shreveport, Shreveport, LA 71103, USA; b Department of Hematology and Oncology, Feist Weiller Cancer Center, Louisiana State University Health Shreveport, Shreveport, LA 71103, USA

**Keywords:** Erythropoiesis, Luspatercept, Thalassemia, Anemia, Myelodysplastic syndrome, Myelofibrosis

## Abstract

Luspatercept is a novel erythroid maturation agent that has emerged as a significant advancement in the management of ineffective erythropoiesis. By targeting the transforming growth factor-β superfamily signaling pathway, Luspatercept enhances late-stage erythroid differentiation. This review provides an in-depth exploration of its mechanism of action, pharmacologic properties, and clinical efficacy across multiple hematologic disorders. We summarize trial outcomes in lower-risk myelodysplastic syndromes, transfusion-dependent β-thalassemia, and myelofibrosis, highlighting improvements in erythroid response and transfusion independence. The article also discusses adverse event profiles and future directions, including ongoing trials and potential expansion of indications. Luspatercept represents a promising targeted erythroid therapy with benefits across several hematologic diseases.

## Introduction

Ineffective erythropoiesis (IE) is a hallmark of various hematologic disorders, including myelodysplastic syndromes (MDS) and β-thalassemia. It is characterized by the inability to produce mature red blood cells (RBCs) despite the presence of increased immature erythroid precursors. Traditional therapeutic approaches, such as RBC transfusions and erythropoiesis-stimulating agents (ESAs) are often limited by associated complications, including iron overload and resistance. Understanding the molecular basis of erythropoiesis has led to the development of novel therapies targeting regulatory pathways. Among these, Luspatercept, a recombinant fusion protein that acts as an erythroid maturation agent, has emerged as a promising treatment option for anemia associated with MDS and β-thalassemia as demonstrated in pivotal clinical trials such as BELIEVE and MEDALIST.

This review aims to provide a comprehensive overview of Luspatercept, including its mechanism of action, clinical efficacy and safety profile, and emerging roles in various hematologic conditions.

## Literature Search and Study Selection

A comprehensive literature search was conducted to identify relevant studies evaluating the mechanism, pharmacology, and clinical efficacy of Luspatercept in hematologic disorders. Electronic databases including PubMed/MEDLINE and Google Scholar were searched for articles published up to March 2026 using combinations of the keywords “Luspatercept,” “erythroid maturation agent,” “ineffective erythropoiesis,” “myelodysplastic syndromes,” “β-thalassemia,” “myelofibrosis,” “real-world data”, “adverse events” and “TGF-β signaling.” Clinical trial registries, including ClinicalTrials.gov, were also reviewed to identify ongoing and recently completed studies. Titles and abstracts were screened for relevance, and full texts were reviewed when appropriate. Priority was given to pivotal phase II and III clinical trials, prospective cohort studies, translational studies elucidating mechanism of action, and key review articles relevant to the therapeutic role of Luspatercept. Abstracts presented at major hematology conferences (e.g., American Society of Hematology (ASH) and American Society of Clinical Oncology (ASCO)) were included when they provided important emerging clinical data not yet available in full publications. Studies were selected based on relevance to the pathophysiology of IE, clinical efficacy, safety profile, and evolving therapeutic indications of Luspatercept, with emphasis on high-quality studies and those with significant clinical impact.

## IE and Its Biomarkers

IE is a central mechanism underlying anemia in disorders such as β-thalassemia, MDS, and myelofibrosis (MF). It is characterized by expansion of erythroid precursors in the bone marrow, impaired terminal maturation, and increased intramedullary apoptosis, resulting in insufficient production of functional RBCs despite increased erythropoietic activity [1, 2].

Pathogenetically, IE involves aberrant activation of TGF-β superfamily signaling. Ligands including growth differentiation factors (GDFs) and activins bind to activin receptors on erythroid progenitors, triggering intracellular SMAD2/3 phosphorylation. This cascade suppresses late-stage erythroid differentiation, leading to accumulation of early precursors and apoptosis of late-stage erythroblasts [1–4].

### β-Thalassemia

In β-thalassemia, IE primarily arises from imbalanced globin chain synthesis, where excess α-globin chains aggregate in erythroid precursors, causing oxidative damage, membrane instability, and apoptosis of erythroblasts. Despite compensatory expansion of erythroid progenitors in response to chronic anemia and elevated erythropoietin (EPO), many precursors fail to mature into functional erythrocytes [5–7].

Several circulating biomarkers reflect IE in thalassemia. Elevated EPO levels reflect compensatory stimulation of erythropoiesis in response to chronic anemia, while soluble transferrin receptor (sTfR) concentrations denote degree of erythropoietic activity and iron demand [8]. Growth differentiation factor-15 (GDF-15) is elevated and suppresses hepcidin, promoting intestinal iron absorption and contributing to iron overload [9]. Erythroferrone (ERFE), secreted by erythroblasts in response to EPO, acts as a negative regulator of hepcidin and is crucial to iron metabolism [8]. Despite strong biological associations, no single biomarker is currently validated for routine clinical monitoring or prediction of therapeutic response.

### MDS

In MDS, IE arises from clonal hematopoietic stem cell disorders characterized by genetic and epigenetic abnormalities that impair erythroid differentiation. Bone marrow is typically hypercellular with erythroid expansion but reduced reticulocyte output. Dysregulation of signaling pathways within the TGF-β superfamily, leading to enhanced SMAD2/3 signaling, suppress terminal erythroid maturation [1, 4, 10].

Recent mechanistic studies have further highlighted the role of aberrant RNA splicing and transcriptional regulation in erythroid failure in MDS. Altered splicing of key erythroid transcription factors such as GATA1 has also been shown to impair erythroid maturation in lower-risk MDS [11]. Additionally, dysregulated GDF11-SMAD signaling has been linked to abnormal splicing events affecting erythroid differentiation pathways, providing further insight into the molecular mechanisms underlying erythroid failure in MDS [12].

### MF

There are several different implicated mechanisms of development of anemia in MF. The most common one is through fibroblastic proliferation induced by certain growth factors released by abnormally expanded megakaryocytes with displacement of medullary erythropoietic tissue. The compensatory hematopoiesis in extramedullary sites is generally less effective [13, 14].

Transforming growth factor-β (TGF-β) is overexpressed in MF and is an important cytokine contributing to fibrosis and megakaryocyte hyperplasia. The role of TGF-β in MF has also been observed in various mouse models, thus making it a potentially viable target for the treatment of MF-related anemia [5].

## Mechanism of Action

Luspatercept is a recombinant fusion protein composed of a modified extracellular domain of the human activin receptor type IIB (ActRIIB) linked to the Fc portion of human IgG1 [3, 4].

Luspatercept acts as a ligand trap by binding selected circulating TGF-β superfamily ligands before they interact with endogenous activin receptors, thereby attenuating SMAD2/3 phosphorylation and promoting terminal erythroid maturation [3, 4]. The fusion protein contains a modified ActRIIB extracellular domain that binds ligands with high affinity, while the IgG1 Fc domain prolongs serum half-life and facilitates systemic exposure [4].

### Ligand targets

Early mechanistic models suggested that GDF11 was the principal ligand mediating erythroid suppression [15], but subsequent studies challenged this hypothesis. In a β-thalassemia mouse model, genetic deletion of Gdf11 did not improve anemia, and the murine analog of Luspatercept (RAP-536) retained efficacy in Gdf11-deficient animals, suggesting that the drug’s activity is not dependent on GDF11 inhibition alone [16, 17]. This finding indicates that Luspatercept likely acts through simultaneous neutralization of multiple TGF-β superfamily ligands, growth differentiation factor-11 (GDF11), growth differentiation factor-8 (GDF8/myostatin), activin B, BMP6, and BMP10, all of which can activate SMAD2/3 signaling through activin receptors [4].

### GATA1 regulation and erythroid differentiation

A 2025 study demonstrated that GDF11-mediated SMAD2 activation can directly influence RNA splicing of the erythroid transcription factor GATA1, a key regulator of erythropoiesis. This leads to skipping of exon 2 in the GATA1 transcript, generating a truncated isoform (GATA1s) which has been found to disrupt erythroid differentiation and contribute to anemia in MDS [11].

Importantly, patients who responded to Luspatercept in clinical studies exhibited higher baseline proportions of GATA1s transcripts, and treatment was associated with a relative reduction in the GATA1s isoform [11].

Preclinical studies have also demonstrated that Luspatercept can restore nuclear availability of GATA1 and its co-activator TIF1γ in erythroid progenitors, further supporting the central role of GATA1-mediated transcription in its mechanism of action [18].

### Effects on the bone marrow microenvironment

Studies in mesenchymal stromal cells derived from patients with MDS demonstrated that Luspatercept reduces SMAD2/3 phosphorylation and restores secretion of stromal factors such as stromal-derived factor-1 (SDF-1) that support hematopoiesis [19]. Pre-treatment of MDS stromal cells with Luspatercept improved the clonogenic potential of co-cultured hematopoietic stem and progenitor cells, suggesting that the drug may partially restore the supportive function of the bone marrow niche [19].

### Additional cellular effects

Treatment with RAP-536 has been shown to reduce intracellular reactive oxygen species in erythroid progenitors and upregulate genes involved in oxidative stress responses, potentially improving survival of erythroid precursors [20]. Notably, these studies did not demonstrate significant changes in clonal mutation burden or variant allele frequency, suggesting that the drug primarily promotes differentiation rather than altering clonal hematopoiesis [20].

### Mechanistic uncertainty

Despite substantial advances in understanding its mechanism, several aspects of Luspatercept biology remain incompletely defined. The relative contribution of individual ligands within the TGF-β superfamily, the molecular basis for differential responses among patients such as ring sideroblast (RS) positive vs. negative patients, and the mechanisms underlying multilineage hematologic responses observed in some studies remain areas of active investigation. Furthermore, although emerging data suggest that molecular features such as SF3B1 mutations and altered erythroid progenitor dynamics may influence therapeutic response, predictive biomarkers have not yet been validated for routine clinical use.

## Pharmacokinetics (PK) and Pharmacodynamics

Chen et al [21] analyzed the PK and exposure response of Luspatercept in MDS using data from 260 patients enrolled in the PACE-MDS, MEDALIST trial, and the PACE-MDS extension studies. The PK was best explained by a one-compartment model with first-order absorption and elimination and time-invariant clearance. Luspatercept exhibited linear and consistent behavior over the dosing range of 0.125 to 1.75 mg/kg. A mean elimination half-life of around 13 days was noted, with an interindividual variability in steady-state exposure (AUCs) of 38%. Key factors influencing apparent clearance (CL/F) and volume of distribution (V1/F) included body weight, age, and baseline albumin levels. Both CL/F and V1/F increased with body weight. Notably, weight-based dosing was shown to reduce variability in drug exposure compared to fixed-dose strategies. Both CL/F and V1/F increased with decreasing albumin. Luspatercept CL/F decreased slightly with increasing age. Since individuals with extreme albumin or age values were projected to have a < 20% variation in Luspatercept exposure for weight-based dosing, the influence of albumin and age on Luspatercept serum exposure seemed less clinically important. Other variables—such as renal or liver dysfunction, EPO levels, transfusion history, or presence of anti-drug antibodies—were found to have minimal or no impact on the drug’s PK. Overall, the model demonstrated reliable predictive accuracy and confirmed the suitability of weight-based dosing for patients with MDS [21].

Further, it was observed that increasing Luspatercept serum exposure increased the likelihood of obtaining an erythroid response (transfusion independence (TI) ≥ 8 weeks) and this response plateaued at doses 1.0–1.75 mg/kg. A slower Luspatercept CL/F highly correlated with a higher likelihood of obtaining an erythroid response. Additionally, as Luspatercept exposure increased, particularly after long-term treatment, the likelihood of developing severe treatment-emergent adverse events (TEAEs) reduced. According to these assessments, the suggested therapeutic dosages (1.0–1.75 mg/kg, every 3 weeks) have a favorable benefit–risk profile [21].

PK, safety, and efficacy data for Luspatercept in individuals with β-thalassemia were obtained from a multi-center, international phase 2 clinical trial (NCT01749540, NCT02268409). The trial included a dose-finding phase with fixed doses ranging from 0.2 to 1.25 mg/kg, followed by an expansion phase starting at 0.8 mg/kg, where doses could be increased up to 1.25 mg/kg depending on individual response. The key PK parameter analyzed was the area under the concentration–time curve (AUC), with clinical endpoints assessing hemoglobin (Hb) rise in non–transfusion-dependent (NTD) patients and transfusion reduction in transfusion-dependent (TD) patients during the initial 15-week period [22, 23].

By mid-2016, data from 89 patients (49 NTD, 40 TD) revealed that Luspatercept displayed linear PK and followed a one-compartment model with first-order absorption and elimination [24]. The drug had a serum half-life of roughly 10 days, an apparent clearance of 0.44 L/day, and a distribution volume around 7.1 L—both of which scale with body weight. Notably, TD patients had approximately 23% lower volume of distribution than NTD patients. Absorption is unaffected by the injection site, and Luspatercept is believed to undergo non-specific protein catabolism [25]. These PK features support a weight-based dosing approach and predict consistent exposure across most patients.

In NTD individuals, higher AUCs were significantly associated with greater increases in Hb (P < 0.01). While many TD patients experienced reduced transfusion needs, a direct exposure-response trend was less pronounced, potentially due to limited exposure range. Median AUC values among responders were similar (∼100 d·µg/mL for NTD and ∼120 d·µg/mL for TD). Simulations projected that a starting dose of 1.0 mg/kg would achieve target AUCs in over 90% of NTD patients and around 50% of TD patients, while a dose of 0.8 mg/kg was insufficient in most cases [24].

### Half-life differences between populations

The modestly longer half-life in MDS patients is primarily due to a larger volume of distribution (9.6 vs. 7.1 L in β-thalassemia), while clearance is similar (≈0.44–0.47 L/day) [25]. Potential contributing factors include differences in age, body composition, and marrow characteristics (expanded erythropoiesis in β-thalassemia vs. clonal dysplastic marrow in MDS) [3, 6]. Despite this, the PK differences are not clinically significant, and the same 3-week dosing interval with weight-based dosing is used for both populations [25].

### Dosing schedule and response

The 3-week interval for Luspatercept injections is based on its half-life and time to steady-state concentrations. Steady state is achieved after three doses (≈9 weeks) in both thalassemia (half-life of 10–11 days) and MDS (half-life of 13 days) [22, 25]. Peak serum concentrations occur around 5–6 days post-dose, and Hb increases can be observed within 7 days of the first dose in patients with low transfusion burden [22, 25]. The greatest Hb rise occurs after the first dose, with smaller incremental increases following subsequent doses [25].

### Dose adherence and interruption

Real-world data on suboptimal adherence to Luspatercept are limited, though the FDA label provides guidance on missed doses. Hb levels return to baseline approximately 6–8 weeks after the last dose, suggesting benefit disappears within this timeframe [25]. The drug is administered every 3 weeks, and if a dose is delayed or missed, it should be given as soon as possible with at least 3 weeks between doses [25]. The largest Hb increase occurs after the first dose (0.75–1 g/dL), with smaller incremental gains on subsequent doses [25]. Real-world studies confirm that responses typically occur within 3–4 months of starting therapy, and many responses are long-lasting while on treatment [26]. However, no specific studies have examined the impact of intermittent dosing or varying degrees of non-adherence on clinical outcomes. Clinical trials show median durations of TI of 30.6 weeks in MEDALIST [3] and 127 weeks in COMMANDS, reflecting continuous dosing; dose interruptions were reported in 20–27% of patients in COMMANDS trial, primarily due to adverse events (AEs), but dose reductions were rare [27].

### AEs

Safety assessments showed that higher AUCs correlated with more frequent grade 2–3 drug-related AEs (P < 0.05), most commonly bone and muscle pain, although these specific events were not associated with serum drug exposure [24].

These results supported starting at 1.0 mg/kg with dose escalation to 1.25 mg/kg as needed.

## Preparation and Administration

Luspatercept is available as a lyophilized powder in single-use vials of 25 and 75 mg for subcutaneous injection, which must be reconstituted with sterile water. To prepare the 25 mg vial, reconstitute it with 0.68 mL of sterile water, resulting in a concentration of 25 mg per 0.5 mL. For the 75 mg vial, use 1.6 mL of sterile water, yielding a concentration of 75 mg per 1.5 mL. Once reconstituted, the solutions can be stored at room temperature (20–25 °C) for up to 8 h [25].

β-thalassemia: Administer 1 mg/kg subcutaneously every 3 weeks, with dose adjustment based on patient response. Discontinue treatment by week 9 if no transfusion benefit is observed [25].

MDS-related anemia: Administer starting at 1 mg/kg subcutaneously every 3 weeks; adjust the dose every 6 weeks, if necessary, up to a maximum of 1.75 mg/kg. Discontinue treatment by week 9 if there is no reduction in transfusion requirement [3, 25].

Self-administration is not permitted—the drug must be prepared and administered by healthcare professionals due to weight-based dosing, reconstitution complexity, storage issues, and pre-dose Hb and blood pressure monitoring requirements [25].

## Clinical Efficacy

The major clinical trials evaluating the efficacy of Luspatercept across hematologic indications are summarized in Table 1 [3, 27–34].

**Table 1:** Summary of Major Clinical Trials of Luspatercept

Trial name	Study type	Year of publication	Disease focus	Primary endpoint	Results, % of Luspatercept responders
BEYOND [34]	Phase 2, randomized, double-blind, placebo	2022	Non–transfusion-dependent β-thalassemia	Hb ↑ ≥ 1 g/dL from baseline for ≥ 2 weeks during weeks 13–24	77.1% vs. 0% in placebo arm; P < 0.0001
BELIEVE [33]	Phase 3, randomized, double-blind, placebo	2020	Transfusion-dependent β-thalassemia	≥ 33% reduction in transfusion burden during weeks 13–24 and reduction in ≥ 2 RBC transfusion units	21.4% vs. 4.5% in placebo arm; P < 0.001
PACE-MDS [28]	Phase 2, open-label	2019	Lower-risk MDS (RS and non-RS)	Hematologic improvement–erythroid (HI-E) per IWG 2006 criteria	Non-RS: 36.4%; RS: 67.7%; Overall: 53.7%
MEDALIST [3]	Phase 3, randomized, double-blind, placebo	2020	Lower-risk MDS with ring sideroblasts	≥ 8 weeks of RBC transfusion independence (RBC-TI) during first 24 weeks	38% vs. 13% in placebo arm; P < 0.001
COMMANDS [29–31]	Phase 3, randomized, open-label	2023	ESA-naive, lower-risk MDS	≥ 12 weeks RBC-TI + mean Hb ↑ ≥ 1.5 g/dL in weeks 1–24	58.5% (Luspatercept) vs. 31.2% (epoetin alfa); P < 0.0001
MEDALIST MDS/MPN-RS-T Substudy [32]	Retrospective *post-hoc* analysis	2020	MDS/MPN-RS-T (retrospective)	≥ 8 weeks RBC-TI during weeks 1–24	64.3% vs. 22.2% in placebo arm; P = 0.028
ACE-536-MF-001 [27]	Phase 2, open-label	2024	Myelofibrosis (MF)	NTD: Hb ↑ ≥ 1.5 g/dL from baseline for 12 weeks; TD: ≥ 12 weeks RBC-TI	NTD: cohort 1: 14%, cohort 3A: 14% TD: cohort 2: 10%, cohort 3B: 26%

Sources: BEYOND trial [34], BELIEVE trial [33], PACE-MDS trial [28], MEDALIST trial [3], COMMANDS trial [29–31], MEDALIST MDS/MPN RS-T sub study [32] and ACE -536 MF-001 trial [27].

### MDS

The earliest studies for Luspatercept action in MDS were performed in mouse models using RAP-536, the mouse version of ACE-536 (Luspatercept). RAP-536 in MDS mice was shown to inhibit SMAD 2/3 signaling in turn decreasing IE and improving anemia. There was a significant rise in RBC count and Hb levels (P < 0.05) [35].

These findings were further studied and confirmed through multiple clinical trials in humans since then including the PACE-MDS phase 2 trial, MEDALIST phase 3 trial, and COMMANDS phase 3 trial.

#### PACE-MDS trial (NCT01749514/NCT02268383)

This is a phase II, multicenter clinical trial evaluating the long-term efficacy and safety of Luspatercept in treating anemia among patients with lower-risk MDS [28].

The study enrolled a total of 58 patients in the 12-week base study. Patients were stratified based on RS status, baseline transfusion requirements (NTD, low transfusion burden (LTB) (< 4 RBC units per 8 weeks), high transfusion burden (HTB) (≥ 4 RBC units per 8 weeks)), pre-treatment serum erythropoietin (sEPO) levels, and presence of SF3B1 mutations.

Patients received subcutaneous Luspatercept every 21 days at doses ranging from 0.125 to 1.75 mg/kg [28]. The primary endpoint was hematologic improvement–erythroid (HI-E), as defined by the International Working Group (IWG) 2006 criteria: 1) HTB patients: Reduction of ≥ 4 RBC units or a ≥ 50% decrease in RBC transfusion requirement over an 8-week period compared to the baseline transfusion burden. 2) LTB patients: Increase in Hb levels by ≥ 1.5 g/dL from baseline for ≥ 14 consecutive days.

In the initial base study, 63% patients receiving higher dose Luspatercept concentrations (0.75–1.75 mg/kg) achieved HI-E versus 22% patients receiving lower dose concentrations (0.125–0.5 mg/kg). Thirty-eight percent of TD patients achieved red blood cell transfusion independence (RBC-TI) for at least 8 weeks [28].

The extension study enrolled 108 patients to evaluate long-term safety and durability of response. The study included 64 patients with RS (RS+) and 44 without RS [36]. The study was primarily designed to evaluate safety, which was adequately assessed given the sample size and median exposure of 315 days. However, the study was underpowered to make conclusions on efficacy comparisons [36].

Secondary analyses showed an overall HI-E rate of 53.7%, with higher responses in RS-positive patients (69%) than RS-negative patients (36.4%) [36]. NTD patients had a 70.6% HI-E response, and 77% of patients with SF3B1 mutations responded. Exploratory analyses also reported multilineage improvement: 33.3% had HI-neutrophil responses and 9.5% had HI-platelet responses [36]. Biomarker analyses suggested that responders exhibited an increased late-to-early erythroid progenitor cell ratio, consistent with Luspatercept’s mechanism of promoting late-stage erythroid maturation.

Baseline EPO levels appeared to correlate with response, with HI-E rates of 76%, 58%, and 43% in patients with EPO < 200, 200–500, and > 500 IU/L, respectively, representing suggestive trends rather than definitive predictive relationships [36].

Luspatercept demonstrated a favorable safety profile over long-term use. The most common TEAEs were fatigue (9.7%), hypertension (6.5%), and diarrhea (6.5%) in patients with RS and headache (11.4%), hypertension (6.8%), and bone pain (6.8%) in patients without RS. One RS patient progressed to acute myeloid leukemia (AML). No new safety signals emerged during the study [36].

##### 1) Strengths and limitations

PACE-MDS provided important early evidence of Luspatercept’s activity in lower-risk MDS across both RS-positive and RS-negative patients, as well as NTD individuals, with durable erythroid responses and a favorable long-term safety profile (median exposure 315 days) [36]. The study also offered mechanistic insights through biomarker analyses and multilineage hematologic activity. However, interpretation of efficacy is limited by the phase II single-arm design with a relatively small sample size (108 patients) and the absence of a placebo control [36]. Furthermore, the heterogeneous patient population—including both TD and NTD individuals as well as previously treated and untreated patients—makes it difficult to draw definitive conclusions regarding efficacy across specific subgroups. Subgroup analyses, particularly in RS-negative patients and higher-dose cohorts, were underpowered, and the primary endpoint focused on safety rather than definitive efficacy [36].

#### MEDALIST trial (NCT02631070)

A phase III, randomized, double-blind, placebo-controlled trial was conducted at 65 sites across 11 countries to evaluate the efficacy and safety of Luspatercept in treating anemia in lower-risk myelodysplastic syndromes (LR-MDS) patients with RS [3].

The study included a total of 229 patients with either very-low, low, or intermediate risk MDS as defined by IPSS-R criteria with ≥ 15% RS or ≥ 5% RS if SF3B1 mutation was present and less than 5% bone marrow blasts [3]. Other key inclusion criteria were patients who were refractory, intolerant, or unlikely to respond to ESAs and patients who had a regular RBC transfusion requirement (≥ 2 units per 8 weeks in the 16 weeks before randomization) [3].

Patients were randomized in a 2:1 ratio to receive either Luspatercept (1.0–1.75 mg/kg subcutaneously every 21 days) (153 patients) or placebo (76 patients). Both the groups were balanced in all their baseline characteristics.

The primary endpoint was RBC-TI for ≥ 8 weeks during weeks 1–24. Important secondary endpoints included: 1) RBC-TI ≥ 12 weeks assessed for both weeks 1–24 and weeks 1–48; 2) HI-E response defined by the IWG 2006 criteria; 3) Increase in Hb levels by at least 1 g/dL; 4) Safety and AE assessment.

Fifty-eight patients (38%) in the Luspatercept group achieved the primary endpoint of TI during weeks 1–24, as compared with 10 (13%) in the placebo group (P < 0.001) [3].

Ninety percent of the Luspatercept responders showed their first response at the starting dose of 1 mg/kg.

The response was inversely proportional to the number of RBC units the patient was requiring prior to the study time period with the greatest response seen in patients receiving less than 4 units per 8 weeks (during the 16 weeks prior to treatment) [3].

TI of 12 weeks or longer (RBC-TI ≥ 12) during weeks 1–24 was seen in 28% patients in the Luspatercept group compared to 8% in the placebo group (P < 0.001). During weeks 1–48, 33% patients in the Luspatercept group showed TI ≥ 12 weeks compared to 12% patients in the placebo group (P < 0.001).

A higher percentage of patients in the Luspatercept group also showed TI for 16 weeks or longer. Twenty-two patients (14%) in the Luspatercept group maintained their response at 1 year. The median duration of the longest continuous response to Luspatercept was 30.6 weeks [3].

Similar trends were seen in erythroid response as well as mean Hb increase of ≥ 1 g/dL with significantly greater number of patients responding in the Luspatercept group than the placebo group during weeks 1 through 24 as well as weeks 1 through 48.

No significant population-level changes in neutrophil or platelet counts were observed during the double-blind period [3]. A dedicated secondary analysis of the MEDALIST trial demonstrated that some patients with baseline neutropenia or thrombocytopenia experienced increases in neutrophil or platelet counts, despite the absence of significant overall population-level changes [37]. This has both positive implications (potential multilineage benefit in cytopenic patients) and safety considerations (potential worsening of thrombocytosis in MDS/MPN overlap syndromes). This particular safety concern has also led to the ongoing phase 2 (NCT05005182) trial of Luspatercept with or without hydroxyurea in MDS/MPN-RS-T patients [38]. However, these multilineage effects have not been associated with increased rates of disease progression, transformation to AML, or adverse safety signals in major trials [3].

The most common AEs that were reported during the trial included fatigue (seen in 27% Luspatercept vs. 13% placebo group patients), diarrhea (22% vs. 9%), asthenia (20% vs. 12%), nausea (20% vs. 8%), dizziness (20% vs. 5%), and back pain (19% vs. 7%) [3]. Grade 3 or 4 AEs were seen in 42% patients receiving Luspatercept compared to 45% patients receiving placebo. Thirty-one percent patients receiving Luspatercept had a serious AE as compared to 30% receiving placebo [3]. A total of 13 patients (8%) in the Luspatercept arm and six patients (8%) in the placebo arm discontinued treatment due to AEs. Overall, the safety profile of Luspatercept was comparable to placebo, with a manageable side effect profile and the findings were consistent with that reported in the phase 2 PACE-MDS study [28, 36].

One patient in each group progressed to higher-risk MDS. AML developed in four patients, including three patients (2%) in the Luspatercept group and one patient (1%) in the placebo group [3].

These results led to the FDA approval of Luspatercept in April 2020 for the treatment of anemia in TD LR-MDS patients with RS.

##### 1) Strengths and limitations

The MEDALIST trial demonstrated a statistically significant improvement in TI with Luspatercept compared with placebo [3]. Long-term follow-up data of the trial [39] showed durable responses, with a median cumulative duration of response of approximately 80 weeks, and a generally favorable safety profile [39]. However, the study population was restricted to RS-positive patients, limiting generalizability to other MDS subtypes [3]. Additionally, the study was not powered to evaluate progression to AML, and a substantial proportion of patients (69.3%) required dose escalation to maximum dose (1.75 mg/kg), while a majority (62%) did not achieve the primary TI endpoint [3].

#### COMMANDS trial (NCT03682536)

A phase 3, open-label, randomized controlled study conducted at 142 sites in 26 countries to compare the efficacy and safety of Luspatercept against epoetin alfa in ESA-naive patients with TD, LR-MDS [29].

The study population included a total of 363 patients with very low, low, or intermediate-risk MDS (per the Revised International Prognostic Scoring System), who were ESA-naive and TD (requiring 2–6 packed RBC units per 8 weeks for ≥ 8 weeks immediately before randomization), with sEPO levels less than 500 U/L [30].

Patients were randomly assigned in a 1:1 ratio to Luspatercept or ESA arm and stratified based on baseline RBC transfusion burden (< 4 units per weeks vs. ≥ 4 units per 8 weeks), endogenous sEPO level (≤ 200 U/L vs. > 200 to < 500 U/L) and presence of RS [29, 30].

Patients in the Luspatercept arm received Luspatercept subcutaneously every 3 weeks at doses ranging from 1.0 to 1.75 mg/kg. Patients in the epoetin alfa group received epoetin alfa subcutaneously once a week, starting at 450 IU/kg with possible titration up to 1,050 IU/kg (maximum total dose of 80,000 IU) [29].

The primary endpoint was achievement of RBC-TI for at least 12 weeks with a concurrent mean Hb increase of at least 1.5 g/dL within the first 24 weeks [29].

The interim efficacy analysis was done for 301 patients who had completed 24 weeks of treatment or discontinued earlier. Eighty-six (59%) of 147 patients in the Luspatercept group and 48 (31%) of 154 patients in the epoetin alfa group reached the primary endpoint (P < 0.0001) [31].

HI-E for ≥ 8 weeks was achieved in 74.1% of patients receiving Luspatercept and 51.3% of patients receiving epoetin alfa (P < 0.0001). Within the first 24 weeks of treatment, RBC-TI for ≥ 24 weeks was achieved in 47.6% of Luspatercept-treated patients compared to 29.2% of epoetin alfa-treated patients (P = 0.0006). Similarly, RBC-TI for ≥ 12 weeks was observed in 66.7% of patients in the Luspatercept group versus 46.1% in the epoetin alfa group (P = 0.0002) [29].

Later the primary analysis of this ongoing trial showed similar findings. A total of 363 patients had been randomized by September 2022. A significantly higher number of patients in the Luspatercept arm (60%) reached their endpoint compared to 35% patients in the epoetin alfa arm (P < 0.0001) [30].

Long-term follow-up has also provided insights into overall survival (OS). At extended follow-up (median ∼29 months), median OS was not reached for the Luspatercept arm, compared with 46.7 months in the epoetin alfa arm (hazard ratio (HR) 0.86; 95% confidence interval (CI) 0.60–1.24), with 3-year OS rates of 63.8% vs. 62.2% and 4.5-year OS rates of 58.9% vs. 41.8%, respectively, favoring Luspatercept [40, 41]. Similar trends toward improved OS were seen across subgroups defined by baseline transfusion burden, RS status, and sEPO levels [41]. These data suggest a potential survival benefit with Luspatercept, although OS was not the primary endpoint of the study and longer follow-up is needed to confirm this observation.

TEAEs of any grade were reported in 92.1% of Luspatercept-treated patients and 85.2% of epoetin alfa-treated patients [29]. Discontinuation due to TEAEs occurred in 4.5% (n = 8) of Luspatercept-treated patients and 2.3% (n = 4) of epoetin alfa-treated patients. Treatment-related AEs (TRAEs) were observed in 30.3% of Luspatercept-treated patients and 17.6% of epoetin alfa-treated patients. Progression to AML was reported in four patients receiving Luspatercept and five patients receiving epoetin alfa. Overall mortality rates were comparable between treatment arms, with 18.0% (n = 32) of Luspatercept patients and 18.2% (n = 32) of epoetin alfa patients experiencing death during or after treatment.

Grade 3 or 4 TEAEs were similar in both arms and included hypertension, anemia, dyspnea, syncope, thrombocytopenia, neutropenia, pneumonia, COVID-19, and MDS. The most common serious TEAEs in both groups were pneumonia and COVID-19 [29].

The safety profiles of both treatments were comparable, suggesting that Luspatercept could represent a new standard of care for this patient population.

Further analyses of the COMMANDS trial have shown the superiority of Luspatercept over ESAs in achieving higher reduction in transfusion burden [42] and a shorter time required to improve health-related quality of life [43]. In a long-term analysis of the trial [44, 45], Garcia-Manero et al reported that 44.5% of patients treated with Luspatercept achieved at least 1 year of RBC-TI, compared to 27.6% of patients receiving epoetin alfa. Additionally, 30.2% of Luspatercept-treated patients maintained TI for over 18 months. These benefits were observed consistently across various subgroups, including patients without RS and those with low baseline EPO levels. The incidence of progression to high-risk MDS or AML remained low and comparable between the treatment arms [44].

##### 1) Strengths and limitations

The COMMANDS trial was the first randomized study directly comparing Luspatercept with the standard-of-care ESA [31]. The study reached the primary endpoint in 58.5% Luspatercept participants vs. 31.2% ESA participants with a P value < 0.0001 [29]. Responses were also more durable, with a median duration of TI of 127 weeks in the Luspatercept arm, and about 44.5% achieving ≥ 1 year of TI [31]. Importantly, the trial included a broader patient population than earlier studies, enrolling both RS-positive and RS-negative patients and demonstrating benefit across several molecular subgroups [31, 44]. It is to be noted however, that the study population was enriched for RS-positive disease (73%) and SF3B1 mutations (59%), which may still limit generalizability to the broader MDS population [30]. The open-label design may also have introduced performance and reporting bias [29, 30]. In addition, the cohort also lacked racial and ethnic diversity, with more than 80% of participants identified as White [30]. Furthermore, longer median treatment exposure with Luspatercept (41.6 vs. 27.0 weeks in ESA arm) complicates direct safety comparisons [29]. Higher rates of AEs such as hypertension, thromboembolic events, and renal injury were observed, although some differences attenuated after adjustment for treatment exposure [31].

#### ELEMENTS-MDS (NCT05949684)

An ongoing phase 3, open-label, randomized study designed to evaluate the efficacy and safety of Luspatercept compared to epoetin alfa in treating anemia among adults with very low, low, or intermediate-risk MDS who are ESA-naive and NTD [46].

Luspatercept has been approved for use in ESA-naive as well as ESA refractory TD LR-MDS patients. The primary aim of this trial is to compare the efficacy of Luspatercept versus epoetin alfa in delaying or preventing the progression to TI in the special patient population of NTD LR-MDS [46].

Important inclusion criteria include sEPO levels ≤ 500 U/L, symptomatic anemia, and baseline Hb ≤ 9.5 g/dL. The primary endpoint is the proportion of participants who become TD during any continuous 16-week period from weeks 1 to 96. As of March 2025, the ELEMENT-MDS trial is actively recruiting participants across multiple international sites [46].

##### 1) Strengths and limitations

This study is uniquely and importantly investigating earlier use of Luspatercept which can potentially reduce chronic disease burden. However, treating NTD patients holds the risk of exposing them to unnecessary AEs without clear benefit.

#### Meta-analysis data

A recent systematic review and meta-analysis by Alhajahjeh et al (2025) [47] integrated data from 20 studies encompassing 3,455 patients with lower-risk MDS, including clinical trials and real-world cohorts, to provide a comprehensive assessment of Luspatercept’s efficacy and safety. The pooled results demonstrated that 8-week TI was achieved in 50.2% of patients, 12-week TI in 57%, and 24-week TI in 35.8%, with HI-E rates of 51.3%. Patients with RS-positive disease or lower baseline transfusion burden derived the greatest benefit, with RBC-TI reaching 72.9% in LTB patients and 38.7% in high transfusion burden patients [47]. Safety analyses confirmed common AEs such as peripheral edema, diarrhea, and fatigue, without new or unexpected signals [47]. While this meta-analysis supports the robustness and generalizability of erythroid responses across diverse populations, several gaps and limitations remain: direct head-to-head comparisons with ESAs or other therapies are lacking, cross-trial evaluations are limited by differences in patient characteristics (e.g., RS status, del(5q), prior ESA exposure), long-term clinical benefits remain unclear, and the influence of molecular profiles (e.g., SF3B1 and other mutations) on treatment response is still poorly defined. Additionally, data on neutrophil and platelet responses remain sparse, the potential for combination regimens has not been systematically explored, and efficacy in RS-negative patients appears limited, highlighting ongoing unmet needs and areas for future investigation [47].

#### Additional studies

Both the COMMANDS and ELEMENT-MDS trials are currently ongoing and data from preliminary analysis of the COMMANDS trial continues to confirm the efficacy and safety of Luspatercept [31, 45].

The efficacy and safety profile of Luspatercept in MDS continues to be studied across the world [48, 49]. The phase 3 LusPlus (NCT05181592) [50] and Maxilus study (NCT06045689) [49] aim to assess the safety and efficacy of using Luspatercept at the highest approved dose of 1.75 mg/kg. Early findings from both of these trials demonstrated a safety profile consistent with previous studies [50, 51]. Moffitt Cancer Center in Florida, USA is currently conducting a study (NCT04064060) to evaluate the long-term safety in patients who have participated in other Luspatercept clinical trials [52]. A phase II trial at MD Anderson Cancer Center (NCT06113302) is evaluating the efficacy of Luspatercept in reducing transfusion requirement in LR-MDS [53]. Kosugi et al, through a phase 2 trial in NTD LR-MDS Japanese patients, concluded that Luspatercept resulted in statistically and clinically significant improvements in Hb levels and may help delay the need for transfusions [54].

Multiple ongoing studies are currently evaluating the broader benefits of Luspatercept across diverse patient populations and in combination with other therapies, as highlighted in the following examples. A retrospective analysis from the MEDALIST trial evaluated the efficacy and safety of Luspatercept in patients with myelodysplastic syndrome/myeloproliferative neoplasm with ring sideroblasts and thrombocytosis (MDS/MPN-RS-T) [32]. The results showed that 64.3% patients with MDS/MPN-RS-T achieved RBC-TI for ≥ 8 weeks during weeks 1–24 compared with 22.2% receiving placebo (P = 0.028) [32]. An ongoing single-arm, open-label phase 2 clinical trial (NCT05732961), is evaluating Luspatercept efficacy in patients with LR-MDS as well as MDS/MPN [55]. Another phase 2 trial (NCT05005182) by Mangaonkar et al is investigating the efficacy and safety of Luspatercept, with or without hydroxyurea, in treating patients with MDS/MPN-RS-T or unclassifiable MDS/MPN with ring sideroblasts (MDS/MPN-U-RS) [38].

A multi-center phase Ib/II study (NCT04539236) is aiming to assess the combination of Luspatercept with lenalidomide to treat anemia in LR-MDS patients without 5q gene deletion [56]. The preliminary results have shown promising results, and a phase II trial is ongoing [56].

The GFM COMBOLA (NCT05181735) is another interesting two-part phase 1/2 study which is evaluating the efficacy of the combination of Luspatercept with ESAs in patients with lower-risk non-RS MDS who have not responded to ESA monotherapy [57]. Results from the dose-determining phase 1 part of the study showed a significant erythroid response of 30% by week 25 with a safety profile that is considered manageable. The ongoing phase 2 part of the study aims to compare the combination regimen with high-dose Luspatercept monotherapy [57]. A similar study by Komrokji et al investigated the efficacy of combining Luspatercept with ESAs in patients who had not responded to monotherapy with either agent alone. A 36% response rate was demonstrated, indicating a potential synergistic effect [58]. Patients that responded better included those with previous response to Luspatercept monotherapy, endogenous sEPO levels < 500 U/L, SF3B1 mutation, lower RBC transfusion burden and patients who had not been previously treated with hypomethylating agent or lenalidomide [58].

### Thalassemia

In β-thalassemia murine models, Luspatercept administration resulted in a dose-dependent increase in Hb levels, reduced IE, and decreased spleen size, indicating improved erythroid maturation and reduced extramedullary hematopoiesis [59]. Additionally, studies on hbbth3/+ mice, a well-established model of β-thalassemia, showed that Luspatercept significantly increased RBC count and hematocrit levels, while also reducing sEPO levels, suggesting an overall improvement in erythropoietic efficiency [60]. These findings were further supported by studies in non-human primates, where Luspatercept treatment led to a sustained increase in Hb concentration without excessive erythropoiesis stimulation, distinguishing it from traditional ESAs [61].

Phase 1–3 trials in humans replicated these benefits and eventually, Luspatercept was FDA approved in 2019 for adults with transfusion-dependent β-thalassemia (TDT) based on the results of a pivotal phase 3 study, the BELIEVE trial.

#### Phase II trial (NCT01749540 and NCT02268409)

A phase 2, open-label, nonrandomized dose-finding study was conducted to evaluate the safety, tolerability, and efficacy of Luspatercept in adult patients (≥ 18 years) with TDT and non–transfusion-dependent β-thalassemia (NTDT). Patients received Luspatercept subcutaneously every 21 days at doses ranging from 0.2 to 1.25 mg/kg. The study consisted of two stages: an initial dose-escalation stage, where patients received up to five doses, and an extension stage, where responders continued treatment at higher doses [23].

A total of 64 patients were enrolled, with 33 classified as NTD (mean hemoglobin < 10.0 g/dL; < 4 RBC units transfused per 8 weeks) and 31 as TD (≥ 4 RBC units per 8 weeks). The median age was 38.5 years (range: 20–62 years), and 48% were female. The primary endpoint of the trial was defined as an ≥ 1.5 g/dL from baseline for ≥ 14 consecutive days (without RBC transfusions) for NTD patients or RBC transfusion burden reduction ≥ 20% over a 12-week period vs. the 12 weeks before treatment for TD. Among NTD patients, 58% achieved this primary endpoint at dose levels of 0.6–1.25 mg/kg. Additionally, 71% of NTD patients achieved a ≥ 1.0 g/dL increase over 12 weeks. In TD patients, 81% experienced a ≥ 20% reduction in transfusion burden, with 72% achieving a ≥ 33% reduction and 63% achieving a ≥ 50% reduction over 12 weeks. Both TD and NTD patients with prior splenectomy had a higher response rate compared to those with intact spleens [23].

Luspatercept was well tolerated, with grade 1–2 AEs being the most common, including bone pain, headache, and injection site reactions. Grade 3 AEs were rare, occurring in 8% of patients, and no treatment-related grade 4 AEs or deaths were reported. Improvements in iron overload, bone mineral density (BMD), and quality of life (QoL) were observed, with significant reductions in liver iron concentration (LIC) among patients on iron chelation therapy [23]. Notably, six patients with chronic leg ulcers achieved complete healing, suggesting potential benefits beyond erythropoiesis modulation.

##### 1) Strengths and limitations

This phase II study demonstrated early clinical activity of Luspatercept in patients with β-thalassemia, showing improvements in hemoglobin levels and reductions in transfusion requirements across both TD and NTD populations [23]. The study included multiple dose cohorts and provided important dose-response data that informed the optimal dosing strategy used in later trials such as BELIEVE trial [23, 33]. However, the study was limited by its relatively small sample size and non-comparative design, which restricts definitive conclusions regarding efficacy [23]. In addition, the heterogeneous patient population and lack of a placebo control make it difficult to fully assess treatment effect across different β-thalassemia subgroups [23].

#### BEYOND trial (NCT03342404)

A pivotal phase 2 clinical trial evaluated the efficacy and safety of Luspatercept in adult patients (≥ 18 years) with NTD β-thalassemia or HbE/β-thalassemia. Eligible participants had received ≤ 5 RBC units in the 24 weeks prior to randomization and had a mean baseline Hb of ≤ 10.0 g/dL. Patients were randomized 2:1 to receive either Luspatercept (1 mg/kg, titrated to 1.25 mg/kg) or placebo (PBO) subcutaneously every 3 weeks for ≥ 48 weeks, with both groups receiving the best supportive care, including occasional RBC transfusions and iron chelation therapy [62].

The primary endpoint was the proportion of patients achieving a ≥ 1.0 g/dL mean Hb increase from baseline over a continuous 12-week interval (weeks 13–24) without RBC transfusions. Secondary endpoints included the proportion of patients achieving ≥ 1.5 g/dL Hb increase, transfusion-free status from weeks 1–24, and improvement in NTD β-thalassemia patient-reported outcome (NTDT-PRO) tiredness and weakness (T/W) scores, which reflect QoL [62].

A total of 145 patients were randomized (96 Luspatercept, 49 PBO), with a median age of 40 years (range: 18–71 years) and 41.7% male participants. The baseline median Hb was 8.2 g/dL (range: 5.3–10.1 g/dL). Among Luspatercept-treated patients, 77.1% (74/96) met the primary endpoint, compared to 0% in the placebo group (P < 0.0001). Response rates were consistent across baseline Hb levels: 1) 72.7% of patients with Hb < 8.5 g/dL achieved the primary endpoint (P < 0.0001 vs. PBO). 2) 82.9% of patients with Hb ≥ 8.5 g/dL achieved the primary endpoint (P < 0.0001 vs. PBO).

Furthermore, 52.1% of Luspatercept-treated patients achieved a ≥ 1.5 g/dL Hb increase during weeks 13–24, compared to 0% in the placebo group (P < 0.0001). Additionally, 89.6% of Luspatercept-treated patients remained transfusion-free at weeks 1–24, compared to 67.3% in the placebo group (P = 0.0013). Improvement in NTDT-PRO T/W scores correlated with Hb increase, with significant improvement observed in patients with baseline Hb < 8.5 g/dL (P = 0.0433) [62].

Luspatercept was generally well tolerated, with grade ≥ 3 TEAEs occurring in 28.1% of Luspatercept patients vs. 24.5% in the placebo group. Notably, no thromboembolic events were reported in either group. Malignant events were only observed in the placebo group, with two cases (diffuse large B-cell lymphoma and hepatocellular carcinoma).

Crossover from placebo to Luspatercept was permitted in the study after unblinding. However, due to lack of long-term data, efficacy endpoints were not reported for crossover group. Safety events in crossover group were similar to the original Luspatercept group [34].

##### 1) Strengths and limitations

The BEYOND study was unique in studying NTDT patients, a population with limited therapeutic options [34]. The study demonstrated a high response rate, with ≥ 1.0 g/dL increase in hemoglobin achieved in 94.8% of patients receiving Luspatercept compared with 22.4% in the placebo arm [34]. Long-term follow-up extending to approximately 4.6 years showed sustained Hb improvement along with durable benefits in patient-reported outcomes such as reductions in fatigue and weakness. However, the phase II design and relatively small sample size limit the generalizability of these findings [34]. Safety considerations included reports of extramedullary hematopoiesis events (9.0% vs. 4.1%), although exposure differences between groups limit direct comparison, and thromboembolic events were observed in a small number of patients (4), all of whom had > 1 baseline risk factors [34].

#### BELIEVE trial (NCT02604433)

A phase 3, randomized, double-blind, placebo-controlled study, evaluated Luspatercept efficacy and safety in TDT. Conducted across 65 sites in 15 countries, the trial enrolled adults (≥ 18 years) with confirmed β-thalassemia or HbE/β-thalassemia who had received 6–20 RBC units within 24 weeks prior to randomization, with no transfusion-free period > 35 days. Patients were randomized 2:1 to receive either Luspatercept (1.0 mg/kg, titrated up to 1.25 mg/kg) or placebo every 3 weeks for at least 48 weeks, alongside best supportive care, including RBC transfusions and iron chelation therapy [33].

The primary endpoint was the proportion of patients achieving a ≥ 33% reduction in transfusion burden from baseline during weeks 13–24, with at least 2 fewer RBC units over this 12-week period. Secondary endpoints included the proportion of patients achieving: 1) A ≥ 33% reduction in transfusion burden during weeks 37–48. 2) A ≥ 50% reduction in transfusion burden during weeks 13–24 and 37–48. 3) Changes in iron parameters, including serum ferritin and LIC.

A total of 336 patients were randomized (224 Luspatercept, 112 placebo), with a median treatment duration of ∼64 weeks. Luspatercept significantly reduced transfusion burden compared to placebo: 1) 21.4% of Luspatercept-treated patients achieved the primary endpoint, compared to 4.5% in the placebo group (P < 0.001). 2) A ≥ 33% reduction in transfusion burden during any 12-week interval was achieved in 70.5% of Luspatercept-treated patients vs. 29.5% of placebo patients. 3) A ≥ 50% reduction in transfusion burden during any 12-week interval was observed in 40.2% vs. 6.3% (P < 0.001).

At week 48, Luspatercept-treated patients had a mean serum ferritin reduction of −348 µg/L (95% CI, −517 to −179), compared to an increase in the placebo group [33].

Luspatercept was generally well tolerated, with bone pain, arthralgia, dizziness, hypertension, and hyperuricemia being more common than in the placebo group. Serious AEs occurred in 15.2% of Luspatercept-treated patients vs. 5.5% in the placebo group. Thromboembolic events were reported in 3.6% of Luspatercept-treated patients (compared to 0.9% in the placebo group), primarily in those with a history of splenectomy and additional thromboembolic risk factors. Treatment discontinuation due to AEs was low (5.4%), and no treatment-related deaths were reported.

Patients in the placebo arm were allowed to cross over to open label Luspatercept after unblinding, enabling evaluation of efficacy and safety in previously untreated patients receiving the drug later in the disease course. In the final long-term analysis, 315 patients received Luspatercept overall, including 92 patients who crossed over from placebo after unblinding [63, 64].

Subsequent long-term follow-up and rollover studies evaluated outcomes in both patients originally randomized to Luspatercept and those who crossed over from placebo. One hundred and twenty-seven out of224 (56.7%) of the original Luspatercept group and 67/92 (72.8%) of crossover patients entered the long-term follow-up study to continue treatment [63, 64]. Importantly, erythroid responses were similar in crossover patients, with approximately 77.2% achieving ≥ 33% reduction in transfusion burden during any 12-week interval, compared with 80.4% in patients initially randomized to Luspatercept [63]. Median treatment duration across the combined studies exceeded 220 weeks (∼4–5 years), demonstrating durable responses with prolonged therapy [3]. Long-term follow-up also showed sustained reductions in transfusion burden and improvements in iron parameters, including decreases in serum ferritin and LIC, in both the original and crossover groups [63].

Safety outcomes were consistent between groups. In the combined BELIEVE and rollover studies, treatment discontinuation occurred in 59.4% of the original Luspatercept group and 47.8% of crossover patients, most commonly due to patient withdrawal rather than AEs, while discontinuation due to adverse events remained relatively low (≈12.5% and 7.6%, respectively) [63]. Importantly, no new safety signals or treatment-related deaths were identified during long-term follow-up, supporting the durability and tolerability of Luspatercept therapy in TDT [33, 63].

##### 1) Strengths and limitations

The BELIEVE trial was a large multinational study [33, 34]. The trial met its primary endpoint in 21.4% of patients receiving Luspatercept compared with 4.5% in the placebo arm (P < 0.001) [33]. Broader clinical benefit was also observed, with achievement of ≥ 33% reduction in transfusion burden during any 12-week interval and significant reductions in serum ferritin levels (−348 µg/L at week 48) [33]. Long-term follow-up data are available up to 156 weeks and demonstrate sustained efficacy across diverse genotypes, including β^0^/β^0^ and HbE/β-thalassemia [64]. However, several limitations should be considered. The primary endpoint response rate remained relatively modest (only 21.4% during the fixed 12-week interval) [33]. While early analysis showed less response in certain genotypes (β^0^/β^0^), further *post hoc* sub-analysis has shown similar response in β^0^/β^0^ subtype although the response differed between splenectomized and nonsplenectomized patients [33, 63]. The study design also relied on investigator discretion for transfusion decisions rather than standardized Hb thresholds, which may introduce variability in outcome assessment [64]. Additionally, long-term comparisons with placebo were limited because patients received placebo for up to 96 weeks but Luspatercept for up to 192 weeks [64]. Safety considerations included a higher incidence of thromboembolic events in the Luspatercept arm (3.6% vs. 0.9%), particularly among splenectomized patients with additional risk factors [33, 64].

#### Meta-analysis data

A recent meta-analysis by Guntupalli et al (2025) pooled data from clinical trials and real-world studies in both TDT and NTDT patients to evaluate the efficacy and safety of Luspatercept [65]. The analysis demonstrated that Luspatercept significantly reduced transfusion requirements, with ≥ 33% reduction in transfusion burden in 74% of TDT patients and 27% achieved ≥ 50% reduction while NTDT patients showed meaningful Hb increases averaging 1.2–1.5 g/dL [65]. Safety outcomes were consistent with prior reports, with the most common AEs being headache, fatigue, and arthralgia, and few grade ≥ 3 events. Major limitations of this meta-analysis include the small number of studies and variability in study design, endpoints, and follow-up durations and overall limited generalizability. The analysis included only one placebo-controlled trial (BELIEVE study) [33, 65].

#### Additional studies

All completed trials (including BELIEVE) enrolled adults ≥ 18 years. Currently, no published human data exist for children. A phase 2 pediatric trial (NCT04143724) is ongoing in children to assess safety, PK, and tolerability. Part A plans to enroll adolescents (12 to < 18 years) in dose-escalation and expansion cohorts for TD and NTD patients. After ≥ 1 year of treatment and safety review, Part B will include children aged 6 to < 12 years [66].

### MF

Anemia in MF is commonly present at diagnosis and studies have shown that about 38% of the patients have symptomatic anemia (i.e., Hb < 10 g/dL) at initial diagnosis and 24% require RBC transfusions [67]. Median survival in patients requiring RBC transfusions at diagnosis has been reported to be 2.6 years compared with 8 years in patients not requiring transfusion [68].

A phase 2 trial in 2023 showed that Luspatercept improves anemia in both primary and secondary MF. The phase 3 INDEPENDENCE study aims to further test Luspatercept against placebo.

#### ACE-536-MF-001 (NCT03194542)

This is a multi-center, open label phase II trial that aimed to evaluate safety and efficacy of Luspatercept for treatment of anemia in MF [27].

The study enrolled 95 patients with MF and categorized them into four cohorts based on transfusion dependence and concurrent ruxolitinib (RUX) therapy. Cohort 1 (n = 22) and 2 (n = 21) patients were not receiving concomitant RUX. Cohort 3A/B patients were receiving stable RUX before and during the study. Cohort 1 and 3A (n = 14) patients were NTD but anemic (≥ 3Hb level results ≤ 9.5 g/dL recorded on different days with no RBC transfusion in the 12 weeks before study period). Cohort 2 and 3B (n = 38) patients were TD (defined as requiring 4 to 12 RBC units during the 12 weeks immediately preceding cycle 1 day 1) [27].

All participants received subcutaneous Luspatercept starting at 1.0 mg/kg (with potential titration up to 1.75 mg/kg) every 21 days. Patients exhibiting clinical benefit at day 169 could continue treatment for 2 years or more (extension treatment period).

The primary endpoint of the study was to assess for anemia response over any 12-week period during the primary treatment phase (weeks 1–24), defined as: 1) For NTD patients: 12 consecutive weeks with Hb increase of ≥ 1.5 g/dL from baseline without transfusions. 2) For TD patients: Achievement of TI for 12 consecutive weeks [27].

It is important to note that the measures of TI or anemia response are not consistent across different studies evaluating anemia.

Key secondary endpoints included achievement of anemia response lasting the entire treatment duration, assessing the maximum duration of anemia response during the entire treatment period, frequency of requiring RBC transfusions, change in Hb levels from baseline, symptom response improvement, and health-related QoL [27].

The anemia response rate in NTD patients was 13.6% in cohort 1 and 14.3% in cohort 3A during the primary treatment period. The response during the entire treatment period (average duration of 2 years) was 18.2% in cohort 1 and 21.4% in cohort 3A. The anemia response rate seen in TD patients was 9.5% in cohort 2 and 26.3% in cohort 3B during weeks 1–24. The response during the entire treatment period was 19.0% and 31.6%, respectively, in cohorts 2 and 3B [27].

It is evident that anemia responses were potentiated by concomitant RUX use (cohort 3A/B) and researchers hypothesized that this resulted due to a synergistic effect of combining JAK1 and JAK2 inhibition by RUX and the erythroid maturation activity of Luspatercept. It was also noted that symptom improvement was more pronounced in the RUX receiving cohorts.

About 27.3% patients in cohort 1 and 50% patients in cohort 3A achieved a mean Hb level increase of ≥ 1.5 g/dL from baseline during the entire treatment period. About 50% TD patients had greater than 50% reduction in their transfusion requirement. There was no significant difference in spleen size that was palpable across all cohorts [27].

Approximately 94% of patients reported at least one AE, with 47% experiencing TRAEs. The most common TRAE was hypertension (18%), managed with medical intervention and did not lead to Luspatercept discontinuation. Fifty percent patients were noted to have a past medical history of hypertension. Further, among patients not receiving RUX, no patients had grade ≥ 3 hypertension events [69].

Diarrhea, dyspnea, thrombocytopenia, and fatigue were some of the other frequently reported AEs. TRAE that led to Luspatercept dose reduction included hypertension, diarrhea, and injection site rash. Nine patients had ≥ 1 TRAE that resulted in study drug withdrawal.

Serious TRAEs were rare; one patient discontinued Luspatercept due to a serious TRAE. Nine on-treatment deaths occurred, none attributed to the study drug. Two (2.1%) patients in cohort 2 transformed to acute leukemia but this was not considered related to treatment. Five thromboembolic events were reported. These events were neither considered related to Luspatercept nor led to Luspatercept discontinuation [69].

While the drug has not obtained FDA approval for MF, it is currently recommended for off-label use by the National Comprehensive Cancer Network (NCCN) to treat anemia in patients with MF.

##### 1) Strengths and limitations

The phase II ACE-536-MF-001 trial demonstrated that clinically meaningful improvements in anemia with Luspatercept and responses were durable with a manageable safety profile. Importantly, Luspatercept did not compromise JAK inhibitor efficacy, with stable spleen size and RUX dosing. However, overall response rates remained modest and variable across cohorts, particularly in patients not receiving RUX. The study was further limited by its small sample size, open-label non-randomized design, and relatively high discontinuation rate due to lack of benefit. Additionally, no clear disease-modifying effects were demonstrated, highlighting the need for randomized studies to better define its role in MF-associated anemia.

#### ACE-536-MF-002/INDEPENDENCE trial

An ongoing global, multi-center phase 3, randomized, double-blind, placebo-controlled study [70] (NCT04717414) evaluating the efficacy and safety of Luspatercept in patients with TD MF-related anemia despite ongoing JAK2 inhibitor therapy.

The study population will include adults (age ≥ 18 years) diagnosed with either primary MF, post-polycythemia vera MF, or post-essential thrombocythemia MF [70].

Other inclusion criteria include patients receiving a stable dose of JAK2 inhibitor therapy for at least 16 weeks prior to enrollment, requiring 4 to 12 RBC units over 12 weeks before randomization.

Important exclusion criteria include anemia due to causes other than MF or JAK2 inhibitor therapy and use of medications with hematopoietic effects such as ESAs, androgens, immunomodulatory drugs (e.g., thalidomide, pomalidomide), or hydroxyurea ≤ 8 weeks before randomization [70].

Participants will be randomized in a 2:1 ratio to receive either Luspatercept (starting at 1.33 mg/kg; titration up to 1.75 mg/kg) subcutaneously, or placebo, every 3 weeks along with best supportive care.

Primary endpoint to be studied is achievement of RBC-TI, over any consecutive 12-week period starting between randomization and week 24 [70].

The study also includes a blinded extension phase after 24 weeks and a post-treatment follow-up will occur 5 years following the first dose of treatment received or 3 years following the final dose received, whichever happens later.

#### ODYSSEY trial

An ongoing phase 2, open-label, multicenter study (NCT06517875) evaluating the efficacy and safety of combining Momelotinib and Luspatercept in patients with TD MF [71].

Several trials have shown the benefits of Momelotinib, a JAK1/JAK2/ACVR1 inhibitor, in patients with MF-related anemia including symptom control, spleen size reduction, and erythroid response leading to its approval for this indication.

The rationale for trials such as ODYSSEY is based on the complementary mechanisms of action of Luspatercept and Momelotinib. Luspatercept functions as a ligand trap for TGF-β superfamily ligands, inhibiting SMAD2/3 signaling and promoting late-stage erythroid maturation [3, 4]. In contrast, Momelotinib uniquely inhibits both JAK1/2 and ACVR1 (ALK2), the latter reducing hepcidin production and improving iron availability for erythropoiesis through the SMAD1/5/8 pathway [72, 73]. Given that anemia in MF results from both IE and inflammation-mediated iron sequestration, targeting these distinct but complementary pathways may enhance erythroid recovery [72, 74].

The ODYSSEY trial aimed to enroll approximately 56 adults (≥ 18 years) with TD primary or secondary MF with 28 JAK inhibitor–naive patients and 28 patients previously treated with JAK inhibitors.

Participants will receive Momelotinib 200 mg orally once daily and Luspatercept as a subcutaneous injection every 3 weeks for up to 24 weeks.

The primary endpoint will be to achieve TI, defined as no RBC transfusions for at least 12 consecutive weeks by week 24. Secondary endpoints will include changes in Hb levels, safety profile, and TI for 24 weeks.

In preliminary results presented at ASH 2025, 14 patients had been enrolled, and early analyses showed stabilization or reduction in transfusion burden in 64% of evaluable patients (7/11) and a reduction in palpable spleen length in 43% (6/14), with manageable safety and no treatment discontinuations due to AEs [75].

#### Additional studies

Data from various other studies conducted around the world have shown a mixed response for Luspatercept in MF. Mayo Clinic (Rochester MN, Arizona, Florida) analyzed data from 37 patients with MF or myeloid neoplasms other than MDS-RS treated with Luspatercept outside the clinical trial setting between July 2020 and May 2022 and concluded that Luspatercept has limited efficacy in MF and myeloid neoplasms other than MDS with RS [76]. About 81% patients in the study eventually discontinued treatment due to lack of response. None of the 10 patients with MF including those receiving concomitant RUX showed an erythroid response. The study limitations included its retrospective nature and small sample size. The researchers also observed that fewer patients in this study had positive RS and SF3B1 mutation compared to other trials like the PACE-MDS which may have contributed to the lower response rates observed [76].

Meanwhile, another small (n = 18) retrospective analysis conducted at multiple centers in China noted significant erythroid response (44% at week 12, 30.8% at week 24, and 50% at the end of follow-up) and Hb increase in patients with refractory anemia related to primary or post essential thrombocythemia/polycythemia vera MF after receiving Luspatercept [77].

While further longitudinal studies are needed to confirm the proposed benefits of Luspatercept in MF-related anemia, real world evidence has shown potential synergistic effect of Luspatercept with JAK2 inhibitors supporting the prior study results [78].

## AEs and Safety Warnings

Luspatercept has demonstrated a favorable safety profile overall across different studies.

Most TEAEs were grade 1–2 and common AEs include fatigue, hypertension, diarrhea, headache, bone pain, arthralgia, and dizziness [3, 29, 34].

Grade 3 or 4 TEAEs were rarely noted. In the COMMANDS trial, grade 3/4 events included hypertension, anemia, neutropenia, pneumonia, COVID-19, and MDS [29]. None of the trials noted death related to Luspatercept.

Most AEs occur early in treatment and are generally low-grade (1–2), with discontinuation due to toxicity in 4.5–8% of patients [3, 33, 64]. Longitudinal data indicate that the incidence of common events like fatigue, bone pain, and dizziness declines over time, independent of dose modifications or interruptions, suggesting tolerability improves with continued therapy [3, 25, 29]. While no studies specifically examine side effects upon resuming therapy after interruption, short delays are unlikely to reset the tolerability profile.

Injection site reactions with Luspatercept are uncommon and generally mild (< 5–7%), including erythema, pruritus, or rash, with no grade ≥ 3 events reported [29].

Progression to AML was reported but not significantly higher in Luspatercept compared to placebo [29].

Despite the theoretic risk of thromboembolic events, such events were rarely noted and one study observed thromboembolism primarily in those with a history of splenectomy and additional thromboembolic risk factors [62].

FDA reports no contraindications of the drug but issues certain warnings and precautions including thrombosis risk, hypertension, extra medullar hematopoietic mass, and embryo-fetal toxicity [25]. Providers are advised to consider thromboprophylaxis and monitoring blood pressure as well as Hb levels regularly. New-onset hypertension or worsening of pre-existing hypertension is managed with anti-hypertensive drugs and Luspatercept discontinuation is rarely indicated. Animal studies have shown adverse outcomes of Luspatercept when administered to pregnant rats and rabbits. Data are not yet available on effects in pregnant women. The warning extends to lactating women as the drug has been detected in animal milk [25].

The drug is currently not studied or approved in pediatric population. FDA explicitly states it is “not recommended” in pediatric populations due to concerning findings in juvenile animal studies, including hematologic malignancies, renal toxicity, and reduced body weight at all tested doses [25].

The safety profile and TEAEs reported in the pivotal MEDALIST, BELIEVE, and COMMANDS trials are summarized in Table 2 [3, 29–31, 33].

**Table 2:** Summary of Safety Outcomes From Key Luspatercept Clinical Trials

Safety outcome	BELIEVE (β-thalassemia) [33]	MEDALIST (MDS with RS) [3]	COMMANDS (LR-MDS, ESA-naive) [29–31]
Most common adverse events (≥ 10%)	Fatigue (27%), headache (26%), bone pain (19%), arthralgia (19%), dizziness (15%), hypertension (10%)	Fatigue (27%), diarrhea (22%), asthenia (20%), nausea (20%), dizziness (14%)	Fatigue (19%), diarrhea (15%), headache (13%), nausea (13%), cough (11%)
Grade ≥ 3 adverse events	∼19% of patients	∼42% of patients	∼26% of patients
Thromboembolic events	∼4% overall (↑ risk in splenectomized)	Rare (< 1%)	∼3% (including deep vein thrombosis and pulmonary embolism)
Hypertension	10% (grade ≥ 3 in ∼1%)	13% (grade ≥ 3 in 3%)	7% (grade ≥ 3 in 1%)
Discontinuation due to AEs	∼5%	∼9%	∼4%
Deaths related to treatment	None reported	One possibly treatment-related (cardiac)	None considered related to treatment
Hypersensitivity reactions	Rare (non-serious rash)	Rare	Rare

Sources: BELIEVE trial [33], MEDALIST trial [3], COMMANDS trial [29–31].

## Real World Data

Real-world evidence demonstrates that Luspatercept is effective across broader and less selected patient populations than those included in clinical trials.

Real world studies in MDS patients have included elderly individuals, heavily pretreated patients, and those with heterogeneous disease features. In a Czech real-world cohort with a median age of 74 years (range 55–95), TI was achieved in 62.7% of patients at 8 weeks and 49% at 24 weeks, with no AEs greater than grade II toxicity reported [79]. This study also included patients with non-standard disease features, including four patients with IPSS-M high-risk disease, who are typically excluded from clinical trials; response rates remained high, with overall response rates of 86% in IPSS-M very low/low-risk groups and 62% in moderate-low groups [79].

Real-world cohorts have further expanded the clinical spectrum of treated patients by including individuals with CMML-0 with RS and other atypical presentations not commonly represented in trials [79]. Additional studies have demonstrated efficacy in heavily pretreated populations. A US single-center study reported 58.1% TI with a median response duration of 58.3 weeks, while multicenter Chinese studies including patients previously treated with ESAs, roxadustat, lenalidomide, or hypomethylating agents showed HI-E responses of 60.9% at 12 weeks, with sustained responses of ∼47–51% during longer-term follow-up in relapsed or refractory disease [49, 80, 81].

The safety profile in real-world settings appears consistent with clinical trials, with discontinuation rates of approximately 4.5–8% due to AEs, and overall favorable tolerability even in older populations [26, 49, 79]. Collectively, these data support the effectiveness and manageable safety profile of Luspatercept across heterogeneous real-world MDS populations beyond the more narrowly defined cohorts of clinical trials.

Similar efficacy was noted in thalassemia patients in real world studies. In a large multicenter Italian real-world cohort of 231 patients treated across 27 centers, clinically meaningful reductions in transfusion burden were observed in routine practice, with a median treatment duration of 272 days; however, 45.9% of patients discontinued therapy during follow-up, most commonly due to lack or loss of response, AEs, or clinical decisions related to inadequate transfusion reduction, reflecting treatment dynamics encountered outside controlled trials [82].

Similarly, a multicenter Greek real-world study including 49 patients reported that approximately 70% achieved ≥ 33% reduction in transfusion burden, while ∼40–50% achieved ≥ 50% reduction, demonstrating substantial hematologic responses across diverse genotypes and baseline transfusion requirements [83]. Additional reports further highlight activity in atypical clinical scenarios. For example, Luspatercept therapy in a patient with TDT complicated by intrathoracic extramedullary hematopoiesis resulted in a marked reduction in transfusion needs with radiologic improvement of the extramedullary mass [84]. Likewise, a recent case series demonstrated hematologic responses in rare dominant β-thalassemia, suggesting potential benefit even in genetic subtypes not represented in pivotal trials [85].

## Special Considerations

### Viral infection

Patients with chronic viral infections (hepatitis B, hepatitis C, or human immunodeficiency virus (HIV)) were systematically excluded from all major Luspatercept trials, including BELIEVE, MEDALIST, COMMANDS, and BEYOND, leaving a significant knowledge gap regarding efficacy and safety in these populations [3, 29, 33, 34]. The exclusion likely reflected early trial concerns about potential hepatotoxicity, immunocompromise, and confounding safety signals. Luspatercept’s mechanism of action is not immunosuppressive, as it promotes late-stage erythroid maturation via TGF-β superfamily ligand binding without affecting lymphocyte function [30, 31, 86]. Clinical trials and post-marketing surveillance have not demonstrated increased infection rates or viral reactivation, though rare hepatic AEs such as hepatitis have been reported [30, 31, 86]. In the absence of direct data, current management should follow general principles for patients with chronic viral infections receiving non-immunosuppressive therapies, including viral screening, monitoring, antiviral prophylaxis for high-risk hepatitis B, treatment of active hepatitis C with direct-acting antiviral (DAAs), and ensuring viral suppression in patients with HIV [87, 88]. Given the prevalence of transfusion-transmitted viral infections in endemic regions, prospective real-world studies are urgently needed.

### Progression risk in low-risk MDS

Available evidence indicates that Luspatercept does not accelerate progression to higher-risk MDS or AML. Across clinical trials and real-world cohorts, progression rates remain low and are consistent with the natural history of lower-risk disease.

In the MEDALIST trial (RS-positive MDS), progression to higher-risk MDS occurred in only one patient per arm, while AML developed in 2% of Luspatercept patients versus 1% with placebo [3]. Extended follow-up showed 13/153 (8.5%) Luspatercept-treated patients progressed to high-risk MDS/AML compared with 5/76 (6.6%) in the placebo group; however, total person-years were longer in the Luspatercept group (401.7 vs. 190.9), suggesting longer time to progression rather than increased risk [34]. In the COMMANDS trial (ESA-naive patients), AML progression was 4% with Luspatercept vs. 4% with epoetin alfa, and higher-risk MDS occurred in 2% vs. 3% respectively. Mortality rates were similar between groups (25% vs. 26%), and incidence rates per 100 person-years confirmed comparable progression risk (AML: 1.76 vs. 2.31; HR 0.82, P = 0.77) [30, 31].

Observational studies in the real world corroborate these low progression rates. In Greek patients, 8.5% progressed to high-risk MDS/AML [26], while a US single-center cohort reported 11% progression [6]. Chinese studies demonstrated conflicting evidence. One study showed 8.7–13% progression over a median 15-month follow-up, while one study noted that Luspatercept “appears to be beneficial in reducing disease progression and prolonging survival” [80, 81].

NCCN guidelines do not express concern about accelerated progression and recommend Luspatercept as a category 1 therapy for MDS with SF3B1 mutations/RS [89]. Standard monitoring with bone marrow evaluation or molecular testing at relapse remains recommended but is not specific to Luspatercept therapy.

## Health-Economic Considerations and Global Access

Despite robust clinical efficacy in MDS and β-thalassemia, widespread access to Luspatercept in low- and middle-income countries (LMICs) is constrained by substantial economic and healthcare system barriers. Annual treatment costs for Luspatercept are estimated between $152,000 and $228,000 per patient in high-income settings. The Canadian Agency for Drugs and Technologies in Health (CADTH) completed a systematic clinical and pharmacoeconomic review of Luspatercept and did not find it to be cost-effective compared with best supportive care (BSC). Per CADTH, estimated incremental cost-effectiveness ratio (ICER) for MDS exceeds $600,000 per quality-adjusted life-year (QALY) and would require price reductions of ∼85% to reach conventional willingness-to-pay thresholds, underscoring challenges even within well-resourced systems [90, 91].

A budget impact analysis in Cyprus projected that reimbursing Luspatercept for β-thalassemia would increase national pharmaceutical expenditure by €21–€25 million over 5 years, highlighting the fiscal strain small health systems face when incorporating high-cost biologics [92].

Such economic pressures are magnified in LMICs, where limited public health budgets, competing priorities, and variable insurance coverage restrict access to novel therapies. In addition to cost, practical barriers such as the need for regular clinic-based administration and monitoring further impede implementation in resource-limited settings. These constraints point to an urgent need for context-specific cost-effectiveness analyses, innovative pricing strategies, patient assistance programs, and policy frameworks to improve equitable access. Moreover, long-term real-world evidence on cost–utility and the balance of clinical benefit versus economic burden in diverse global populations remains sparse, representing a key gap for future research.

## Future Directions

Future research directions for Luspatercept should focus on earlier intervention in disease course, higher starting doses, combination strategies, expansion to additional patient populations, and biomarker-driven patient selection. The COMMANDS trial suggests that Luspatercept should be evaluated earlier in the disease course, potentially as first-line therapy, and at higher starting doses to maximize response rates [31]. Current evidence shows Luspatercept is effective in ESA-naive patients, but optimal timing and dosing strategies require further refinement [31, 93].

Combination approaches also represent an important area of investigation, including regimens with ESAs for synergistic erythroid effects [57, 58], JAK inhibitors such as RUX or Momelotinib in MF [27, 71], hypomethylating agents in higher-risk MDS [94] and with novel agents targeting complementary pathways (e.g., SGLT-2 inhibitors [95], hepcidin modulators [96]). Ideal sequencing in combination regimens is another potential area of future study.

Future studies should also explore expansion to additional patient populations, including higher risk MDS. Preclinical evidence also suggests potential benefit in sickle cell disease, where Luspatercept and its murine analog RAP-536 have demonstrated improvements in anemia and reductions in vaso-occlusive complications in experimental models [97, 98].

Further elucidation of Luspatercept mechanism of action across disease subtypes may be beneficial. Understanding whether it affects other hematopoietic lineages, such as neutrophils and platelets, its effects on iron metabolism and hepcidin regulation, which may influence both anemia and iron overload complications could significantly improve and expand clinical use.

In parallel, identification of predictive biomarkers beyond SF3B1 mutation status will be essential to refine patient selection and better understand which patients derive the greatest benefit from therapy. Finally, ongoing and planned studies are needed to clarify long-term outcomes beyond anemia and symptom improvement. Long-term studies could help assess durability of response, potential disease-modifying effects, the impact of Luspatercept on progression to AML and OS. It would also be interesting to know more about the optimal duration of therapy and management of loss of response. Moreover, longer follow-up could provide a more comprehensive understanding of the safety profile, including the incidence of thrombo-embolic events.

## Data Availability

The authors declare that data supporting the findings of this study are available within the article.
